# Be Just

**Published:** 1878-06-20

**Authors:** 


### BE JUST.

When called, in an emergency, to see the case of a brother practitioner in his absence, avoid, either in language or manner, anything calculated to injure or reflect upon his management of the case. By all means, avoid detailing your successful cures of similar cases. This is an unfair and insinuating method of seeking indirectly to weaken the confidence of the friends in the attending physician by showing your own superior success or skill in like cases. "Let another man praise thee, and not thyself."

